# Current Practice Patterns and Educational Needs of Rheumatologists Who Manage Patients with Rheumatoid Arthritis

**DOI:** 10.1007/s40744-014-0004-5

**Published:** 2014-09-26

**Authors:** Terry Ann Glauser, Eric M. Ruderman, Dale Kummerle, Sheila Kelly

**Affiliations:** 1 CE Outcomes LLC, 107 Frankfurt Circle, Birmingham, AL 35211 USA; 2 Feinberg School of Medicine, Northwestern University, Evanston, IL USA; 3 Bristol-Myers Squibb, Plainsboro, NJ USA

**Keywords:** Biologic agent, Guidelines, Practice pattern, Prescribing behavior, Rheumatoid arthritis, Rheumatology

## Abstract

**Introduction:**

As the therapeutic landscape for rheumatoid arthritis (RA) continues to change, it is relevant to examine current treatment patterns among rheumatologists. The purpose of this study was to identify attitudes and practices of US rheumatologists with respect to RA.

**Methods:**

Nine-hundred and one US-practicing rheumatologists were sent electronic invites (via email or fax) to participate in a case-vignette survey in April 2013. All respondents were currently practicing rheumatology and seeing at least one RA patient per week. The survey examined current attitudes, existing knowledge, management choices and perceived barriers in the management of RA. Data collection stopped once 125 responses were received.

**Results:**

Approximately half of the 125 respondents were very familiar with current clinical practice guidelines for RA diagnosis and management. There was no consensus on which validated tools to use when assessing RA severity, with 54% using Physician Global Assessment and 34% using Disease Activity Score 28 at initial assessment. Most respondents (74%) used methotrexate (MTX) as initial therapy for a newly diagnosed RA patient. Eighty-six percent of respondents would add a tumor necrosis factor inhibitor (TNFi) when MTX alone could not control RA. There was no consensus on which treatment should be used when a TNFi is ineffective. The majority of respondents (66% of respondents) would prescribe TNFis indefinitely in patients with continued response. If a patient was in stable remission on MTX and a TNFi, respondents were most likely to maintain this regimen (53% of respondents); a notable minority (43%) would lower the MTX dose. When prescribing biologics, respondents were most concerned with infection; infection was considered a very significant barrier to biologic use. Although 98% of respondents indicated that they personally educate patients about RA, only 42% provide written material.

**Conclusions:**

The lack of consistency in responses suggests that rheumatologists may benefit from continuing medical education on; clinical practice guidelines; the most recent evidence for management of patients in remission; the use of biologic agents after infection; and management of patients with RA and comorbidities.

**Electronic supplementary material:**

The online version of this article (doi:10.1007/s40744-014-0004-5) contains supplementary material, which is available to authorized users.

## Introduction

More than 1 million individuals in the United States are living with rheumatoid arthritis (RA) [1]. Strong evidence now suggests that early diagnosis and aggressive treatment alter the natural history of RA [2]. Treatment is usually initiated with a synthetic, non-biologic, disease-modifying antirheumatic drug (DMARD) to decrease symptoms, limit joint damage, and improve long-term outcomes [3, 4]. As a well-tolerated, once-weekly oral agent, methotrexate (MTX) is the cornerstone of many treatment regimens and usually the initial DMARD selected for mild, moderate, or severe disease [3, 5]. Over the last decade, several new and potent DMARDs have been approved by the US Food and Drug Administration for the management of RA [6]. These agents, biologic DMARDs, block the effects of cytokines or of immune effector cells or their cell-to-cell interactions [7]. Use of these agents is generally reserved for patients who have failed or are resistant to non-biologic DMARDs, or who have a high level of disease activity at the time of diagnosis and features of poor prognosis [8].

As the therapeutic landscape for RA continues to change, it seems especially relevant to examine treatment patterns for RA to understand how US rheumatologists approach the management of the disease. Several investigators have recently used database information to retroactively elucidate rheumatologists’ practice patterns. Based on an electronic health record review, Adhikesavan et al. [9] evaluated the performance of 15 rheumatologists who practice in an integrated health care delivery system. The rheumatologists’ practices were compared with six quality indicators established by the American College of Rheumatology (ACR). These investigators found that three of the indicators, RA DMARD use, intervention if RA became worse, and MTX risk discussion, were met for high percentages of patients (94, 85, and 87% respectively of the 1,062 patient records reviewed). Percentages of patient records meeting the indicators were lower for RA core data set (69%), MTX baseline studies (41%), and MTX follow-up studies (46%). Because this study focused on a single health care system, the authors proposed to address quality gaps by redesigning the process used to manage patients with RA. Curtis et al. [10] analyzed data from the Consortium of Rheumatology Researchers of North America (CORRONA) to try to determine the impact of rheumatologists’ RA treatment preference on the selection of treatment regimen, independent of patient characteristics. The investigators identified biologic naïve RA patients enrolled in CORRONA who were newly starting therapy with either an anti-tumor necrosis factor (TNF) agent or with a DMARD from 2001 to 2008. In addition, based on the data for the patients’ rheumatologists from the previous calendar year, the authors determined the prescribing patterns of each rheumatologist with respect to anti-TNF agents. The investigators concluded that physician preference is an important factor in whether patients are prescribed anti-TNF agents [10]. Bagheri and Wallace analyzed data from a Southern California pharmacy database, Rx Biotech, to describe prescribing trends for RA biologic therapies by 231 rheumatologists in that region from 2008 to 2010 [11]. They found that anti-TNF agents as a class were the predominant choice of therapy (92.6%) for RA patients seen by private practitioners. Further, the use of etanercept, adalimumab, or infliximab as a proportion of all biologic agents prescribed to RA patients decreased from 93.6 to 75.7%, while two newer agents, golimumab and certolizumab, accounted for 17% of new RA treatments [11]. Harrold et al. [12] used data from the CORRONA registry to evaluate rheumatologists’ prescribing patterns before and after the 2008 publication of the ACR RA treatment recommendations. These investigators found that publication of the guidelines did not significantly change management of patients with active RA [12].

To support the most relevant and effective educational activities for rheumatologists throughout the US, there must be an understanding not only of the practice gaps related to standards of care, but also the readiness of US rheumatologists to change their practice decisions, and the barriers and challenges they face in managing their patients. The information gathered through prospective assessment allows for the delivery of more effective and tailored educational activities based on specific audience needs in order to reduce barriers to care. Furthermore, the information gathered in this type of assessment can be used as a baseline to accurately and objectively measure the outcomes and effectiveness of future educational initiatives. This national study was conducted to prospectively evaluate current US rheumatologists’ practice patterns with regard to the diagnosis and management of RA, familiarity and agreement with guidelines for classification and treatment of RA, and methods of patient communication.

## Methods

A case-vignette survey was developed to examine rheumatologists’ attitudes toward, knowledge and decisions about, and barriers to, the management of RA. The survey consisted of three patient case vignettes—a newly diagnosed patient, a patient with progressive disease and a patient in remission. There were 15 multiple choice questions and 4 Likert scale questions. Invitations for the online survey were distributed to a random sample of US-based practicing rheumatologists by email and fax in April 2013. Criteria for inclusion in the sample were that the respondent must be currently practicing rheumatology and see at least one patient with RA per week. Invitations to participate were sent to 901 US rheumatologists based on information available in the Annual American Medical Association (AMA) Physician Characteristics and Distribution US report, 2011 version; data collection was stopped when 125 surveys were completed. To adequately power the survey, 125 responses were collected, providing a margin of error of ±8% at a confidence interval of 95%. When adequate numbers of surveys were taken, data collection was stopped.

All statistical analyses for the survey data were completed with PASW Statistics 18 (SPSS, Chicago, IL). Descriptive statistics were used to summarize survey responses.

### Compliance with Ethics Guidelines

This study does not contain any new studies with human or animal subjects performed by any of the authors.

## Results

### Respondent Demographics

Respondent demographics are shown in Table 1. The respondent population varied from the national population of rheumatologists as represented in the AMA report in that the study population contained significantly more males and more solo practitioners; all other demographics were nationally representative. Respondents were experienced rheumatologists, with a mean of 28 years since medical school graduation, and seeing, on average, 33 patients per week with RA (range 5–80 RA patients per week).

**Table 1:** Demographic characteristics of survey respondents

	Rheumatologists (*n* = 125)		
	*N*	Sample (%)	US average (%)
Patients seen per week with RA, mean (SD)	33 (25)	–	N/A
Years since medical school graduation, mean (SD)	28 (9)	–	N/A
Gender (% male)	98	78	61
Trained in US	89	71	72
Practice location			
Urban	47	38	N/A
Suburban	71	57	N/A
Rural	7	5	N/A
Present employment			
Solo private	41	33	16
Group private	68	54	44
Non-private (government, academic, or other)	16	13	40
Major professional activity			
Direct patient care	123	98	82
Other	2	2	18

Based on the Annual American Medical Association Physician Characteristics and Distribution US report, 2011

*SD* standard deviation

### Survey Responses

#### RA Management and Treatment Guidelines

When asked about their familiarity with different RA management guidelines, 58% of respondents said they were very familiar with the 2012 update of the 2008 American College of Rheumatology (ACR) recommendations regarding the use of DMARDS and biologic agents [8], while 54% reported that they were very familiar with the 2010 RA European League Against Rheumatism (EULAR) or ACR classification criteria, and 39% said they were very familiar with the EULAR recommendations [13] for managing RA with synthetic and biological disease-modifying antirheumatic drugs (Table 2). Despite this, almost all respondents agreed with the guideline-recommended classification criteria for RA (Table 2). However, 23% disagreed with the guideline recommendations for DMARD therapy in patients with early RA, and 31% disagreed with the guideline recommendation regarding tapering biologic DMARDS for patients in persistent remission.

**Table 2:** Respondent familiarity with RA guidelines

	(*n* = 125)	
	*N*	%
(A) How familiar are you with the following guidelines for the management of RA?		
2012 update of the 2008 ACR recommendations for the use of DMARDs and biologic agents in the treatment of RA		
Very familiar	73	58
Somewhat familiar	47	38
Not familiar	5	4
2010 RA classification criteria: an ACR/EULAR collaborative initiative		
Very familiar	68	54
Somewhat familiar	53	42
Not familiar	4	3
EULAR recommendations for the management of RA with synthetic and biological DMARDs (2010)		
Very familiar	49	39
Somewhat familiar	60	48
Not familiar	16	13
(B) Please specify your agreement with the following statements		
The classification criteria for RA include joint involvement, serology (rheumatoid factor and anti-citrullinated protein antibody), acute phase reactants (CRP and ESR), and duration of symptoms		
Agree	118	94
Disagree	7	6
Patients with early RA who have moderate or high disease activity and poor prognostic features should be started on DMARD combination therapy (including double and triple therapy)		
Agree	96	77
Disagree	29	23
If a patient is in persistent remission after having tapered glucocorticoids, one can consider tapering biological DMARDs, especially if this treatment is combined with a synthetic DMARD		
Agree	86	69
Disagree	39	31

*ACR* American College of Rheumatology, *CRP* C-reactive protein, *DMARD* disease-modifying antirheumatic drug, *ESR* erythrocyte sedimentation rate, *EULAR* European League Against Rheumatism, *RA* rheumatoid arthritis

#### Diagnosis and Ongoing Evaluation

A total of 18 different indices were used to assess RA disease severity at diagnosis, during treatment, or both (Table 3). Physician global assessment (PGA) was most commonly used by respondents, followed by the Disease Activity Score 28 (DAS 28), Routine Assessment of Patient Index Data 3 (RAPID3), Clinical Disease Activity Index (CDAI), and multi-biomarker disease activity test (VectraDA); approximately one-quarter (26%) used less common methods. Notably, the five most common measures were almost always used both at diagnosis and during treatment, and there was a high correlation for use of PGA, DAS28, and RAPID3 for both phases of management.

**Table 3:** Measures used to assess RA disease severity at diagnosis and during treatment, and consistency of their use

(*n* = 125)	Used only at diagnosis		Used only during treatment		Used at diagnosis and during treatment		Used at any point		Correlation between use at diagnosis and during treatment
	*N*	%	*N*	%	*N*	%	*N*	%	
PGA	5	4	10	8	63	50	78	62	0.759**
DAS28 score	5	4	3	2	38	30	46	37	0.857**
RAPID3	7	6	3	2	31	25	41	33	0.808**
CDAI	5	4	1	1	20	16	26	21	0.845**
Vectra biomarker score	6	5	4	3	5	4	15	12	0.505**
Other*	1	1	14	11	17	14	32	26	0.661**

*CDAI* clinical disease activity index, *DAS28* Disease Activity Score 28, *PGA* physician global assessment, *RAPID3* routine assessment of patient index data 3

* Other includes: *HAQ* Health Assessment Questionnaire, *TJC* total joint count, *VAS* visual analog scale; *CRP* C-reactive protein, *CBC* complete blood count, *PGA* patient global assessment, PE, *ACR/EULAR* (American College of Rheumatology/European League Against Rheumatism) remission criteria, *QCRP* quantitative C-reactive protein, *RAPID5* routine assessment of patient index data 5, *SDAI* simple disease activity index, *SJC* swollen joint count, *VASm* Visual Analogic Scale during movement

** Significant at 0.01 level

##### Disease Management

Three patient scenarios were presented to the survey respondents to assess their knowledge and practice patterns.

#### Managing a Newly Diagnosed Patient with RA

Respondents were presented with a patient scenario of a 34-year-old woman with 8 weeks of pain in her hands and wrists and an hour of morning stiffness, but no rash, diarrhea, or low back pain. Over-the-counter (OTC) naproxen provided some pain relief. She had tenderness and swelling of the left wrist, 2 metacarpophalangeal (MCP) joints on each hand, 2 proximal interphalangeal (PIP) joints on each hand, a strongly positive rheumatoid factor (RF), and an erythrocyte sedimentation rate (ESR) of 46. Methotrexate was selected by 74% of respondents as first-line long-term therapy. A minority of respondents would initiate treatment aggressively using triple DMARD therapy (MTX, hydroxychloroquine and sulfasalazine) or a TNF inhibitor (TNFi); 9 and 6%, respectively. Another small group of respondents elected to treat with either hydroxychloroquine alone (10%) or prescription-strength non-steroidal anti-inflammatories (NSAIDs) (2%), regimens that have little or no effect on the underlying disease process.

##### Patient with RA No Longer Controlled by MTX

A case was presented of a 42-year-old man, diagnosed with RA at age 38 years, who had been well controlled with weekly oral MTX 20 mg and folic acid and who had increased pain/swelling in both hands and feet; 2 h of morning stiffness; bilateral wrist synovitis; synovitis of multiple MCP, PIP, and MTP joints; RAPID3 score of 16.3; and a DAS28 score of 5.2. Most respondents (86%) would add a biologic agent to the MTX regimen, 7% would switch to subcutaneous MTX, 2% would add hydroxychloroquine and sulfasalazine, and 6% would choose another strategy.

Respondents were informed that this patient had developed an infection (prostatitis). When asked about the proper time to restart a TNFi for this patient after a single infection, 86% rheumatologists reported that they would do so immediately, while 8% would wait until the patient has a flare of RA, 3% would never restart the TNFi, and 3% selected other.

##### Patient with RA in Remission on a MTX and TNFi Regimen

Another patient scenario, 45-year-old woman with RA in remission on MTX and TNFi, was presented to respondents; two-thirds (66%) would continue a TNFi indefinitely for a patient who is in remission (DAS 28 score of 2.3) while taking MTX and a TNFi; however, 12% said that they would stop the TNFi ≤1 year after the patient achieved remission, and 13% would stop 1–2 years after achieving remission. Nine percent of respondents were unsure as to how long this patient should remain on the TNFi.

When respondents were given a selection of five possible therapeutic changes that they could make in the regimen for a patient whose RA has been well controlled by MTX and a TNFi for 2 years, 53% would not change the regimen, 43% would reduce the dose of MTX, 2% would reduce the dose of TNFi, 1% would discontinue the TNFi, and 1% would discontinue the MTX.

Rheumatologists were evenly split over what to do when a TNFi was ineffective. Forty-eight percent of respondents would try a different TNFi, while 49% would opt for a biologic agent from a different class, 1% would not start another agent, 1% would start a non-biologic DMARD, and 2% selected other. A difference was found between rheumatologists in solo practice versus those in group practice, with 63% of those in solo practice opting for a biologic agent from another class while 41% of those in group practice reported that they would do so.

#### Biologic Therapy: Knowledge and Attitudes

**Fig. 1:**
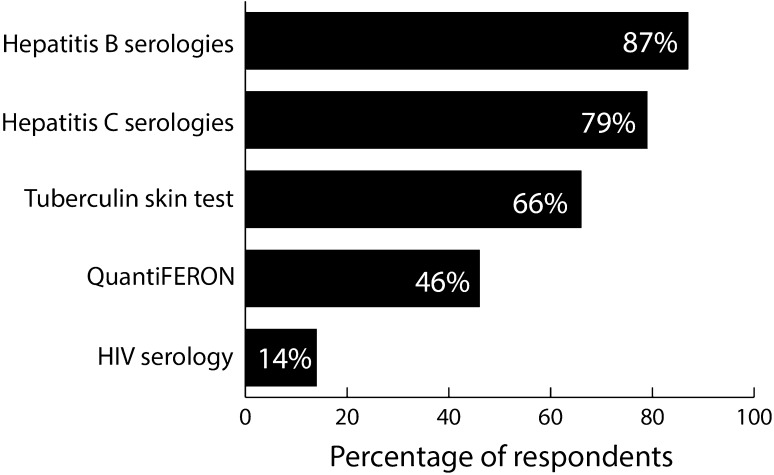
Tests routinely ordered prior to starting a patient with rheumatoid arthritis (RA) on biologic therapy for the first time. Respondents were able to “select all that apply”

Prior to starting a biologic therapy, most respondents (87%) would order hepatitis B serologies, and nearly all would order a test for tuberculosis (TB) with either a tuberculin skin test or QuantiFERON (66 and 46%, respectively; Fig. 1). Hepatitis C serologies would be obtained by a large majority (79%) of respondents, and a small percentage (14%) would test for HIV.

**Fig. 2:**
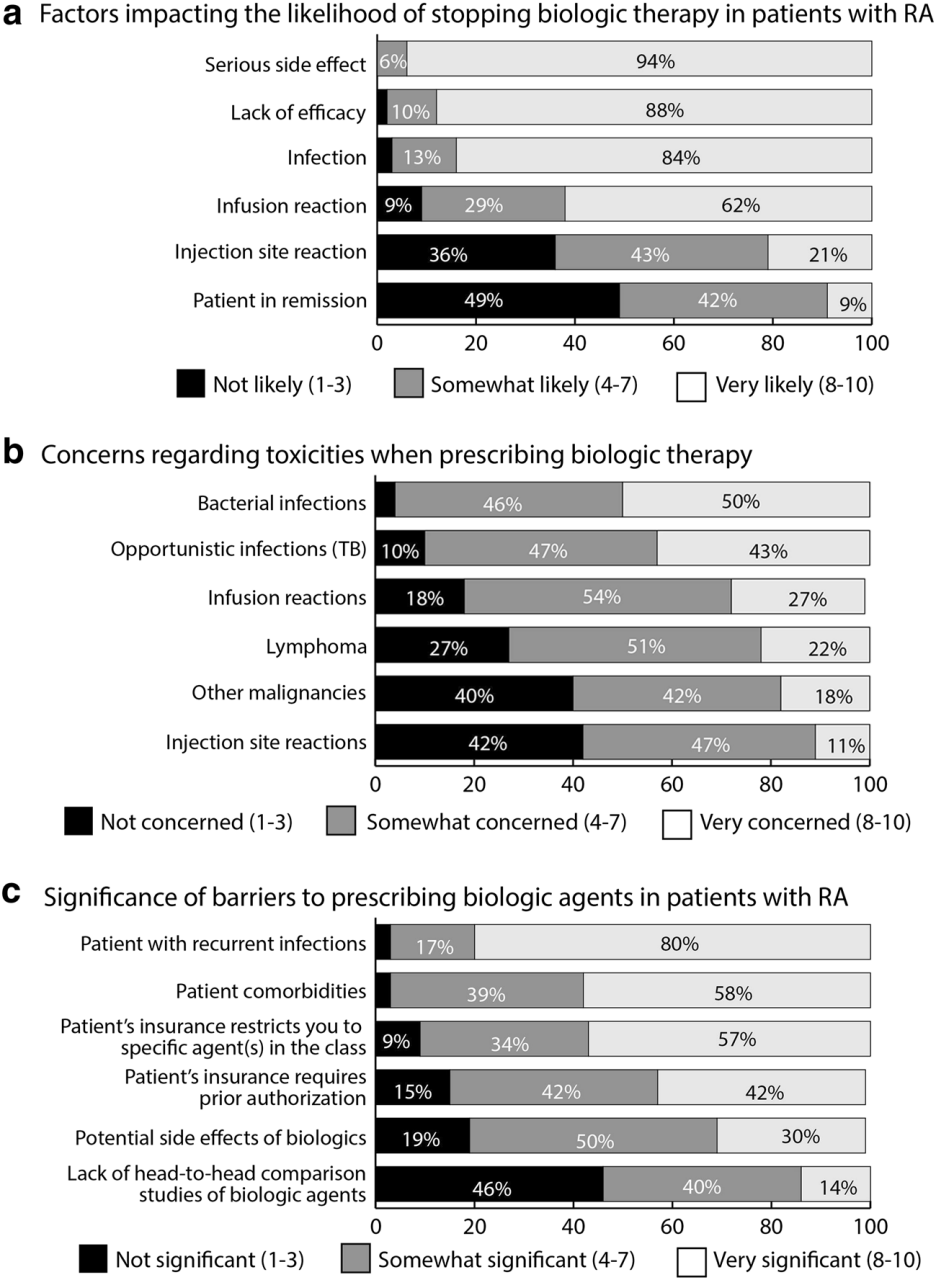
Attitudes toward biologic therapy for patients with rheumatoid arthritis (RA), including: **a** likelihood of stopping a biologic therapy in certain situations; **b** concerns about toxicity of biologic agents; and **c** barriers to prescribing biologic agents in patients with RA. Respondents were asked to rate each question on a 10-point Likert scale

Most respondents would be very likely to stop biologic therapy for a patient with RA because of serious side effects, lack of efficacy, or infection. In addition, approximately two-thirds (62%) said they would be very likely to stop biologic therapy for an infusion reaction and 21% would be ‘very likely’ to stop for an injection site reaction. Only 9% said they would be very likely to stop a biologic therapy because the patient was in remission; however, 42% said they would be ‘somewhat likely’ to do so (Fig. 2a).

When asked about their level of concern regarding potential toxicities of biologic agents, half of respondents were very concerned about bacterial infections, and 43% were very concerned about opportunistic infections, including TB. A quarter or fewer of respondents were very concerned about infusion reactions, lymphoma and other malignancies (27, 22 and 18%, respectively; Fig. 2b).

Recurrent infections were considered a very significant barrier to prescribing biologic agents to the large majority (80%) of respondents. Patient comorbidities and insurance restrictions that limit physicians to prescribing a specific agent or agents in the class were also regarded as very significant barriers (Fig. 2c).

#### Patient Communication

Almost all (98%) respondents indicated that they provide the initial education to their patients about RA and RA medications through personal discussions. Less than half (42%) of respondents said they would provide written materials about RA to newly diagnosed patients, and 50% would provide written materials about RA medicines. Only 15% of respondents would refer patients to websites to learn about RA, and even fewer (6%) would do so to help educate patients on RA medicines. A nurse or physician assistant would provide a patient with initial education about the disease of RA for 10% of respondents and 6% of respondents have a nurse or other individual discuss the management of RA with patients.

**Fig. 3:**
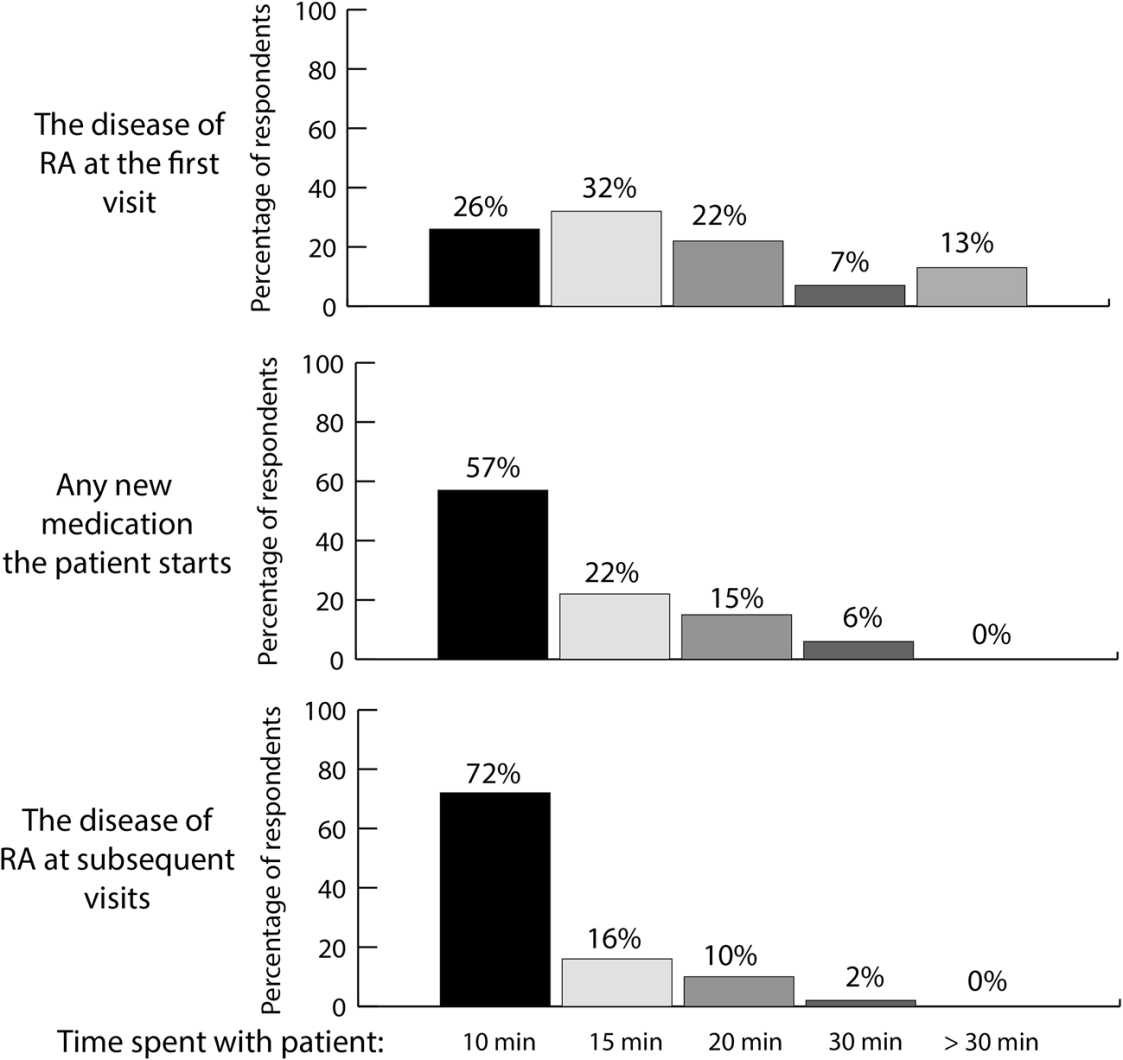
Amount of time spent by respondents and/or their office staff educating patients about rheumatoid arthritis (RA) during first and subsequent visits, and about any new medication prescribed

The majority of respondents spend less than 20 min engaged in educational conversations with their patients, regardless of the subject matter (Fig. 3).

## Discussion

Approaches to the management of RA continue to evolve based on an increased understanding of its pathogenesis and comorbidities and with the development of new agents to address these issues. In the past decade, many highly effective DMARDs with a variety of mechanisms of action have been developed. To address the availability of these new agents, both the ACR and EULAR have recently updated their RA management guideline documents. As knowledge about RA increases, and as novel agents to treat it become available, it is important to assess rheumatologists’ practice patterns with respect to managing the disease. This includes elucidating rheumatologists’ knowledge of and attitudes about new DMARDs and about society guidelines for using them. Gathering information on barriers that may prevent the use of these agents can help focus educational strategies for overcoming them. However, few recent studies have been published on this topic. This study adds to the available information and helps to inform future education for rheumatologists.

Less than two-thirds of survey respondents said they were very familiar with the 2012 ACR RA guideline update and less than half were very familiar with the EULAR RA guidelines. Despite this, most respondents in the current study elected to treat a newly diagnosed RA patient using MTX monotherapy. This is consistent with the older 2008 ACR guidelines, which state that MTX or leflunomide monotherapy can be used for patients with all disease durations and for all degrees of disease activity [14]; the 2012 ACR guideline update does not distinguish between DMARDs for use as monotherapy in newly diagnosed patients with low disease activity and for moderate or high disease activity with the absence of poor prognostic features. The 2010 EULAR guidelines (which were in place when the survey was conducted) and the 2013 update to the EULAR guidelines [15] (subsequently published in October 2013) suggest MTX as initial therapy; indeed, the recommendations note “MTX is considered the anchor drug in RA.”

For a patient with RA for whom MTX monotherapy was no longer sufficient, most respondents would start the patient on a TNFi, consistent with both the 2012 ACR RA guidelines and the 2010 EULAR guidelines; of note, the 2013 EULAR guidelines have broadened to include other biologic DMARDs (abatacept, tocilizumab, and under certain circumstances rituximab) in addition to TNFis. The lack of knowledge of recent guideline revisions demonstrated by respondents suggests recent guideline updates may be an important area for additional medical education of rheumatologists. In addition, subsequent to this survey, the results of two research studies demonstrated that use of triple therapy (MTX, sulfasalazine, hydroxychloroquine) is not inferior to the use of MTX and etanercept, either as first-line therapy or as step-up after failure of MTX monotherapy [16, 17]. Future education may include information about this alternative management strategy.

This study uncovered a lack of consensus among respondents about the management of patients who have stable disease while on MTX and TNFis. When asked about how long patients should remain on TNFis, two-thirds of respondents would continue the TNFi indefinitely but one-quarter would stop the TNFi within two years. However, when asked a more general question, how (if at all) they would change therapy for a patient in remission on MTX and a TNFi, just over half would make no changes while 43% would reduce the MTX and not the TNFi. There is currently a lack of data to inform rheumatologists’ practices in this regard, and it remains an area of ongoing research. The recently published PRESERVE trial (NCT00565409) found that, for patients with RA who had been on MTX and etanercept therapy, those randomized to either conventional or half doses of etanercept in combination with MTX were significantly more likely to maintain low RA disease activity compared with those randomized to MTX alone (etanercept therapy stopped) [18]. Conversely, another recently published trial involving an early RA population, OPTIMA (NCT00420927) found that a higher proportion of trial patients initially treated with a TNFi and MTX achieved stable low disease activity compared with those treated with MTX monotherapy; however, once stable, outcomes were the same whether the TNFi was continued or withdrawn [19]. Many rheumatologists may elect to reduce the dose of MTX because patients don’t feel well when taking it; however, biologic DMARDs are more effective when given in combination with MTX, although the minimal dose of MTX necessary to achieve this effect is not known. In addition, there is ongoing research into the cardiovascular benefits of MTX monotherapy; whether this information can be extrapolated to the use of MTX in combination with biologic DMARDs and whether cardiovascular benefit is related to disease control rather than a specific agent are areas for future research. This is an area where rheumatologists will likely need ongoing education as the results of investigations continue to be published.

Respondents’ attitudes about biologic therapy also reveal areas where there is a need for further education. The majority of rheumatologists would perform hepatitis B and C serology testing, which may influence agent selection [8]. Most would appropriately stop biologic therapy because of serious side effects, lack of efficacy, or infection. However, two-thirds of respondents would stop an RA patient’s biologic therapy because of an infusion reaction and one-fifth would stop biologic therapy if the patient had an injection site reaction. In addition, a majority of respondents thought that recurrent infections and patient comorbidities were very significant barriers to prescribing biologic agents. These are areas where further education may mitigate the barriers to using biologic agents.

Patient education about RA is crucial to promoting adherence to treatment regimens. Although 98% of respondents say they personally educate patients about RA, most respondents spend less than 20 min engaged in educating patients about RA at any visit. In addition, less than half of respondents provide patient materials about RA, refer patients to specific websites about RA, or have their nurse or physician assistant discuss RA with patients. Although physician education cannot address the lack of time available to spend educating patients, providing rheumatologists with resources for patient education (e.g. printed handouts and links to patient-appropriate websites) would form a useful part of an educational program.

### Limitations

This study used a case-vignette survey as a surrogate measure of rheumatologists’ skills and knowledge and did not attempt to verify any information with chart audits or direct observation of practice. However, the use of case vignettes (as compared with chart review and standardized patients) has been shown to provide valid and reliable data on clinicians’ actual practice patterns [20]. The three clinical scenarios that were used in this study do not cover the full spectrum of RA patient scenarios. Future studies should investigate other situations, patient types, and comorbidities in order to be more inclusive of the practice patterns used in the US. The study population contained significantly more male respondents and solo practitioners than the population of rheumatologists listed in the AMA 2011 report, which could have affected responses. There may have been a responder bias based on rheumatologists’ willingness to respond to online surveys, time availability, or other responder or non-responder characteristics. Finally, the small honorarium offered to complete the study and limiting the survey to the first 125 responses could have established a selection bias in rheumatologist responses.

## Conclusion

Although the results of this study indicate that the respondents were not very familiar with current RA guidelines, it did highlight they *are* providing guideline-congruent care. We found disagreement on how to manage a patient in remission amongst respondents; however, evidence for any particular strategy is lacking. Ongoing clinical trials may provide direction in the future. In addition, these rheumatologists are concerned about infection in patients taking biologic agents, management of infusion reactions and injection site reactions, and managing patients with comorbidities. Some of these challenges can be addressed through continuing medical education. Knowledge gaps highlighted by this study, and therefore, potential areas for improved education in rheumatology, include: (1) EULAR and ACR recommendations for classification and treatment of RA; (2) most recent evidence for management of patients in remission; (3) use of biologic agents after infection; and (4) management of patients with RA and comorbidities.

## Electronic supplementary material

Below is the link to the electronic supplementary material.

Supplementary material 1 (PDF 187 kb)
