# Supplementary material for: Current Practice Patterns and Educational Needs of Rheumatologists Who Manage Patients with Rheumatoid Arthritis
Source: Rheumatol Ther. 2014 Sep 26;1(1):31–44. doi: 10.1007/s40744-014-0004-5 (PMC4883259; doi:10.1007/s40744-014-0004-5)
Supplement: Supplementary file 1 — Supplementary material 1 (PDF 187 kb) [file 40744_2014_4_MOESM1_ESM.pdf]

Rheumatology and Therapy
OPEN ACCESS
PEER REVIEWED SUMMARY SLIDE

- Half or fewer of the 125 US-practicing rheumatologists surveyed online were very familiar with the American College of Rheumatology (58% 2012 update on use of DMARDs) and European League Against Rheumatism (54% classification; 39% management) recommendations regarding classification and management of rheumatoid arthritis (RA) patients and one quarter or more disagreed with guideline recommendations for management.
- There is no consensus among rheumatologists on which validated tools to use to assess RA severity and more than one measure is employed for ongoing disease activity assessment.
- There is no consensus on management of patients for whom TNFi therapy is ineffective.
- Rheumatologists reported infections and comorbidities to be very significant barriers to prescribing biologic agents for patients with RA.
- There is a continuing unmet need for education among rheumatologists to address the treatment barriers and knowledge gaps identified.

This summary slide represents the opinions of the authors. Sponsorship for this study was funded by Bristol-Myers Squibb. For a full list of acknowledgments and conflicts of interest for all authors of this article, please see the full text online. Copyright © The Authors 2014. Creative Commons Attribution Noncommercial License (CC BY-NC).
